# Obstetrics

**Published:** 1860-03

**Authors:** 


### OBSTETRICS.

1. On Fœtal Auscultation. Letter from *Dr. Francis Adams* to the Editor of the *Medical Times and Gazette.*—Sir: From the statements which have been made under the present head in this journal, it would appear, contrary to my expectation, that there is such a discrepancy in the views of the stethoscopists regarding the sounds detected by auscultation of the heart and of the uterus, that I consider it necessary to determine, if possible with precision, what may be assumed as the generally admitted facts of the case, before attempting to settle those points about which a considerable difference of opinion prevails, and which I am anxious to establish.

The following, then, would appear to be acknowledged and regarded by all the authorities, from Laennec downwards, as the ratio which subsists between the pulse and the sounds of the heart, both in adults and in new-born children.

Every arterial pulse at the wrist has synchronous with it an impulse in the cardiac region, and every impulse is accompanied by two sounds: first, by a loud, and more prolonged sound; and, secondly, by a feeble, and more abrupt sound. Pulse and impulse are recognized by the touch; the sounds by the sense of hearing. Many people, indeed, find some difficulty in detecting the second sound, even in the adult state, but these are dismissed with a scornful notice by such distinguished stethoscopists as the late writer on fœtal auscultation in this journal. We may assume it, then, as being generally conceded that in the adult state the pulse and impulse range from about 70 to 80, and the cardiac sounds from 70 to 80 double sounds, or from 140 to 160 single sounds. At birth, the highest authorities agree that the pulse is about 140, and consequently the cardiac sounds must be about 280; that is to say, 140 strong and 140 feeble sounds. These are generally denominated the "tic-tac" sounds by the authorities; the "tic" meaning the strong and the "tac" the feeble sounds. In utero no one pretends that generally either the one or the other is less frequent than at birth; and consequently we may venture to assume as admitted that at this period the "tic-tac" sounds, taken together, should range from 260 to 280 per minute.* Whenever, then, an auscultator in the abdominal regions detects regularly such a low number of single sounds as 160, there can be no question that these are to be referred not to the fœtal, but to the maternal heart; at all events, most certainly not to the former. Whether, indeed, the fact be absolutely and unquestionably as stated by the celebrated auscultator in the far North, we shall not pretend to affirm or deny, but this point settled, and the inference is irresistible that these 160 single sounds cannot have proceeded from the fœtal heart. No, no; this is the standard of the adult, and not of the fœtal heart. And, by-the-way, it may be that this is the true explanation of the mistake committed by the learned auscultator in the case of spurious pregnancy related by Dr. Simpson, who may have fallen into the same mistake as our Northern auscultator in confounding the average standard with that of the fœtal heart. We think, then, the question is, in so far, set at rest, that granted 160 single sounds can be regularly detected in auscultation of the pelvic region, no one has any right to infer therefrom the existence of a living fœtal heart in that quarter.

But, it will here be asked, what if about 140 double or tic-tac sounds can be detected per minute in the pelvic region, is not this an absolute and unmistakable mark that a living fœtal heart must exist within? Dr. R. Fergusson, in his Pre-

---

* The writer referred to above (*Medical Times and Gazette*, November 5) makes the singular averment that "Dr. Adams is the first who affirmed that from 130 to 160 double beats could be distinguished." Now this I do not affirm, but I do say that the authorities, from the first writer on it perhaps in this country (see Cyclop. of Med., p. 242) down to the latest authority, Dr. Fergusson, all speak of the fœtal sounds being double, and of excessive frequency; and the latter mentions "the tic-tac sound repeated 140 times per minute." Surely 140 brace of grouse mean 280 birds all the world over! When this writer talks of 60 to 80 maternal pulsations in the minute, surely he who contends so strongly for the distinctness of the second sound of the heart, does not mean to say that this is the amount of the adult sounds. He does not mean surely that there are 30 loud and 30 feeble sounds, but 60 loud and 60 feeble ones. Altogether, however, he evidently confounds beats with sounds. But sounds are double the number of beats.

paratory Essay to Dr. Gouch's work "On the Most Important Diseases of Women," lately published by the New Sydenham Society, answers this question in the negative. In the first place, "it is often not found;" and even when discovered it is no certain mark of pregnancy, as the following case proves beyond controversy: A poor girl was violated: her belly enlarged; and she was supposed to be pregnant; and, no wonder, seeing the tic-tac sound could be heard 140 times in a minute. Yet it turned out to be a serous collection, as was ascertained by tapping with a trocar. It appears, therefore, that whether the allotted number of sounds be regarded as single or double, in neither case are they any certain mark of pregnancy.

We must now say a few words respecting what is usually called the *placental soufflet;* and here again we must come to an understanding regarding the general facts involved in this question.

Bellows sounds, or *bruits de soufflet,* (sounds which have not inaptly been compared to those of a locomotive just about to start on a journey,) are generally admitted to be produced more especially by the pressure of the stethoscope on enlarged vessels, whether arterial or venous, but also not unfrequently, as I myself can attest, by muscular contraction, and the passage of air through an internal tube, such as the trachea in the neck, or the intestinal canal in the abdomen. Altogether these soufflets are admitted to be very fallacious guides in diagnosis; for what physician is there of experience who has not known very many cases in which vascular tumors have been thus diagnosed as aneurisms, and *vice versa?* Few can have had many opportunities of seeing abdominal tumors, and not met with cases in which they were accompanied with strong *bruits de soufflet.* (See Cyclopedia of Medicine, vol. iii. pp. 484, 485.) Dr. Meigs thus expresses the result of his experience in this matter: "As to the value of the *bruit de soufflet* in the diagnosis of pregnancy, it has been found that the earlier opinions were erroneous; and I believe there are few well-informed physicians to be now met with who give it even the smallest portion of their confidence in the unfacile discriminations that they are sometimes charged to make. It is not to be doubted that the sound is produced by the rush of blood in vessels, and, in my opinion, sustained by a very long practice in obstetric auscultation, it depends upon the motion of the blood in the iliacs and hypogastrics. I have certainly heard the same sound after delivery as before the child was born, and I have heard it as dependent upon pressure by tumors within the abdomen. Hence I have not the least confidence in it as an object in obstetric auscultation." (*Obstetrics,* p. 240.) Dr. Fergusson, in the work quoted above, expresses himself to the same effect in the strongest terms. He has known it produced by pelvic tumors in cases of putrid fœtuses and morbid placenta; hence he holds that "it affords no diagnostic mark as to fœtal or placentary development."

After all this, what can we think of the Northern auscultator who, on the contrary, affirms that "scarcely any one at the present day entertains a doubt that in almost every case of pregnancy after the seventh month, both the pulsations of the fœtal heart and the *placental soufflet* can, with a moderate degree of care, be readily distinguished," and "that there is no possibility of a mistake being committed here;" for that "the sound of the fœtal heart can by no stretch of imagination, no flight of fancy, no exercise of faith, no intensity of impression, be confounded with any other: it is a sound *per se*"?

I am confident the writer of these sentences stands alone, at the present day, in holding the strong opinions here announced; and I shall only beg leave to add that I trust he does not reduce them to practice himself, nor encourage the youth of the profession to do so. For most assuredly if the young practitioners of the obstetric art regard it as a rule that whenever, in attending cases of protracted labor, they cannot with moderate care detect a soufflet and 160 sounds in the pelvic regions, they are quite warranted in dealing with the child as a dead body, and in applying the perforator to its head; they will soon become familiar with sights which will fill their hearts with sorrow and cover their faces with shame; sights which I forbear to describe, although I have both heard and read of such cases; but I feel grateful to Providence that albeit I am denounced as the holder of heterodox doctrines in obstetrical stethoscopy, I never was condemned to witness any such in my own practice.

With regard, then, to fœtal auscultation, I think the following points have been satisfactorily determined:—

1. That the cases of spurious pregnancy related by Dr. Simpson, in which eminent auscultators fancied they could detect the double sounds of a fœtal heart when there were none present, and the various other instances of a similar character related above, all go to prove that this process of diagnosis is not at all to be relied upon as a test of pregnancy.

2. That the leading facts of the case are so differently stated by different individuals as to put it beyond doubt that these statements must have been much modified by previous impressions and modes of faith.

3. That since soufflets are often heard in the case of pelvic tumors, after delivery, and when the fœtus is putrid and the placenta morbid, they cannot be regarded as placental, nor as indicative of pregnancy at all.

4. That the sounds detected in the uterine region, unless double, cannot have been cardiac, nor, unless double the arterial pulse of the fœtus, can they have been connected with its heart, consequently that such an amount as from 140 to 160 single sounds cannot be referred to the fœtal heart.

5. That one of the authorities for fœtal auscultation admits candidly that even this number of 140 double or tic-tac sounds is often not present in pregnancy; and, on the other hand, that it is sometimes present when there is no fœtus in utero.

6. That the fœtal heart is so surrounded by a large mass of dense maternal structures and blood-vessels, and by the solid limbs and organs of the child, that it seems next to incredible that any sound emitted by it could ever reach the ear of an auscultator.

7. That the whole system of fœtal auscultation originated soon after the dawn of general auscultation, when men's minds were excited by the love of novelty, and warped by many erroneous impressions and mistaken modes of thinking, and has since been mainly upheld by authority.—(*Med. Times & Gaz.*, Dec. 17, 1859.)

2. A POLYPUS-SHAPED DESMOID OF NEARLY THREE POUNDS WEIGHT EXTIRPATED BY BREAKING UP. By *Dr. Gustav Simon*, etc.—This case of desmoid polypus is interesting, both on account of the structure of the growth and the operation for its removal. A lady, aged fifty-one, had been known to carry a polypoid growth for four years, but had refused operative measures. The pelvis seemed almost filled by the tumor; and six months before Dr. Simon's treatment an attempt had been made to seize the tumor with the obstetric forceps, in order to bring it within reach of other instruments. This attempt was abandoned. It seemed to have had the effect, by compression, of lessening the bulk of the tumor, and the voiding the bladder and rectum became easier for a time. The patient was much emaciated, and confined to bed. The tumor quite filled the pelvis; no os uteri could be found. By palpation between the vagina and the abdomen an immense tumor was made out, having its apex at the vulva and basis at the umbilicus. Near the umbilicus was a smaller tumor the size of a hen's egg, which was made out satisfactorily to be the uterus. It being considered impossible to bring down the huge tumor with the obstetric forceps or with Museux's instrument, the lower part of it was held with the latter instrument, and funnel-shaped pieces were cut out, so that its circumference was lessened; then the obstetric forceps was applied, and by compression with this the polypus was drawn partly out of the vagina. On account of the weakness of the patient, this was done without chloroform. After two hours' painful operation, four large pieces were cut out of the apex of the tumor by the scissors, with inconsiderable bleeding. But soon the hemorrhage increased so as to bring on syncope. The hand was forced up to the vagina, and a stalk being felt, the tumor was dragged down by the hand so as to bring the stalk externally. This was transfixed by a needle carrying a double thread, tied on both sides, and cut through. The patient had recovered in twelve days. The following is a summary of the description of the tumor: The investing substance was of uterine tissue which had been distended by the growth within it of desmoid balls projecting out of the cervical cavity, and becoming enormously hypertrophied. These desmoid structures were of all sizes, from that of a pin's head to that of a child's head. On the surface of the mucous membrane covering the tumors, especially on the part where the broad basis was, were numerous vesicles filled with gray transparent mucous contents, and between these were equally numerous funnel-shaped openings, of various sizes, leading into roundish, smooth-walled, empty sacs. These open sacs and closed vesicles, which lay in the deepest strata of the mucous membrane, and not seldom communicated, were nothing else than the enlarged, perhaps multiplied, mucous crypts, and the enlarged, partly burst and partly unburst, ovula Nabothi of the normal cervical mucous membrane. The second constitutive part of the tumor consisted in desmoid balls of various sizes. They were mostly round, of very thick, concentrically-disposed fibrous layers, strongly resisting section by the knife. They were surrounded by the investing uterine substance, and loosely united by connective tissue.—(*Monatsschr. für Geburtsk.*, July, 1859; *Brit. and For. Med.-Chirur. Rev.*, January, 1860.)

